# Characterising West Nile virus epidemiology in Israel using a transmission suitability index

**DOI:** 10.2807/1560-7917.ES.2020.25.46.1900629

**Published:** 2020-11-19

**Authors:** José Lourenço, Robin N Thompson, Julien Thézé, Uri Obolski

**Affiliations:** 1 Department of Zoology, University of Oxford, Oxford, United Kingdom; 2 Mathematical Institute, University of Oxford, Oxford, United Kingdom; 3 Christ Church, University of Oxford, Oxford, United Kingdom; 4 Joint Research Unit Epidemiology of Animal and Zoonotic Diseases (EPIA), INRA, VetAgro Sup, Saint-Genès-Champanelle, France; 5 School of Public Health, Tel Aviv University, Tel Aviv, Israel; 6 Porter School of the Environment and Earth Sciences, Tel Aviv University, Tel Aviv, Israel

**Keywords:** West Nile virus, WNV, mosquito, suitability, transmission, climate

## Abstract

**Background:**

Climate is a major factor in the epidemiology of West Nile virus (WNV), a pathogen increasingly pervasive worldwide. Cases increased during 2018 in Israel, the United States and Europe.

**Aim:**

We set to retrospectively understand the spatial and temporal determinants of WNV transmission in Israel, as a case study for the possible effects of climate on virus spread.

**Methods:**

We employed a suitability index to WNV, parameterising it with prior knowledge pertaining to a bird reservoir and *Culex* species, using local time series of temperature and humidity as inputs. The predicted suitability index was compared with confirmed WNV cases in Israel (2016–2018).

**Results:**

The suitability index was highly associated with WNV cases in Israel, with correlation coefficients of 0.91 (p value = 4 × 10^− 5^), 0.68 (p = 0.016) and 0.9 (p = 2 × 10^− 4^) in 2016, 2017 and 2018, respectively. The fluctuations in the number of WNV cases between the years were explained by higher area under the index curve. A new WNV seasonal mode was identified in the south-east of Israel, along the Great Rift Valley, characterised by two yearly peaks (spring and autumn), distinct from the already known single summer peak in the rest of Israel.

**Conclusions:**

By producing a detailed geotemporal estimate of transmission potential and its determinants in Israel, our study promotes a better understanding of WNV epidemiology and has the potential to inform future public health responses. The proposed approach further provides opportunities for retrospective and prospective mechanistic modelling of WNV epidemiology and its associated climatic drivers.

## Introduction

Global trends that favour the establishment of mosquitoes and movement of infectious hosts (humans and animals) are promoting the geographical expansion and epidemic activity of mosquito-borne viruses [1-4]. Important examples of these trends are the recent Zika virus epidemic, which had severe public health consequences in Central and South America [5-8], the yellow fever vaccination crisis in African countries [9] and (re)emergence of yellow fever virus in Brazil [9,10]. Other examples of mosquito-borne viruses experiencing recent surges include chikungunya, Mayaro, Usutu, Spondweni, Oropuche and West Nile viruses [1,3,11].

West Nile virus (WNV) endemicity is maintained in a transmission cycle between mosquitoes and birds, with human and equine spillover outbreaks caused by the broad host tropism of the mosquito species involved (*Culex* spp.) [12]. Contrary to birds, mammals are inefficient amplifier hosts because they develop low viraemia [13,14]. Currently, there are neither licensed vaccines nor a particular antiviral treatment available for human WNV infection [15]. Most human infections are believed to escape passive surveillance systems owing to their mild clinical nature, although a small proportion of people may develop encephalitis with a substantial risk of death. Because of the latter, WNV is considered one of the most important agents of viral encephalitis globally [15,16].

During the 20th century, WNV outbreaks were mostly reported in Israel and African countries [17,18]. After its introduction to New York City in 1999, WNV quickly became endemic in Canada and 48 states in the United States [14]. Concurrently, epidemic activity increased in Russia, Europe and the Middle East [17-19]. The epidemiological behaviour of WNV in Europe is spatially heterogeneous but in 2018, the continent experienced its largest outbreak [19,20]. The reasons for differences in the spread of WNV between countries and continents are not well understood, but the relationship between climate and mosquitoes is accepted as a key factor since seasonal changes strongly dictate mosquito population dynamics and virus–mosquito transmission efficiency [12,21-25].

Israel is a critical study region for WNV epidemiology with a rich epidemic history dating back to 1951 [17,26,27], a current human seropositivity of 11% [28,29], widespread spatial distribution of *Culex* spp. [28], in particular *Culex* *pipiens* and *perexiguus* Theobald [30], and a unique location between Africa, Europe and Asia working as transit zone for inter-continental bird migration routes [17,28,31,32]. Similarly to continental Europe, Israel experienced a large increase in reported WNV cases in 2018, amounting to 136 cases, 1.6 times higher than in 2016 (n = 86) and 3.4 times higher than in 2017 (n = 40). 

In this study we set out to characterise in detail the transmission potential of WNV in Israel using a mosquito-borne suitability measure termed the index P [33,34]. Suitability measures are an increasingly common, data-driven practice in mosquito-borne disease epidemiology, relying on meteorological variables as drivers of mosquito population dynamics and virus–mosquito transmission efficiency [23,24,35]. We have validated the suitability index P in previous research, by successfully characterising the transmission potential of *Aedes*-borne pathogens, such as dengue virus in Brazil and Myanmar, Zika, dengue and chikungunya viruses in Honduras [33,36], and Zika virus in the Amazon region [34].

Here, we used a combination of temperature and humidity time series as input for the index P estimation, to explain the timing and duration of WNV outbreaks in Israel between 2016 and 2018.

## Methods

### The suitability index P

Index P is a suitability index for the transmission potential of mosquito-borne viruses. Briefly, the basic reproduction number of a mosquito-borne virus R_0_ can be formulated as R_0_ = MP, with M being the average number of adult female mosquitoes per host and the index P the transmission potential of a single adult female mosquito in an immunologically naive host population. M is generally unknown and difficult to quantify, while the index P is a mathematical expression based on mosquito, virus and host parameters that can be obtained from dynamic transmission models (in this case, from [7]). The relationships between meteorological variables and mosquito and viral parameters – e.g. how the extrinsic incubation period varies with temperature or the oviposition rate varies with humidity – are part of the formulation of the index P. These relationships have been estimated in experimental studies (e.g. [37]). Mathematical details can be found in our recent publication introducing the theory, practice and free R package that estimates the index P [33].

### Index P adapted to the West Nile virus zoonotic cycle

To estimate index P, there is a need for prior biological knowledge related to the host, virus and mosquito species, such as the mean and variation of mosquito and host lifespans, or of viral incubation periods. By setting these values, the user calibrates index P to a particular host–pathogen system. In this study, we defined priors for a generalised bird host, *Culex* spp. and WNV virus (Table). Compared with our previous research (e.g. [33,36].) based on human hosts, *Aedes* spp. and e.g. dengue virus, some of the largest differences in priors relate to shorter incubation periods of WNV and lifespans of the mosquito and bird hosts. The index P, as adapted here, is an estimate of WNV transmission potential in the zoonotic cycle. It is meant to serve as a proxy for the potential of WNV spillover events to the human population. It should be noted that no changes to the intrinsic mathematical formulation of the index P were required in this study (see Discussion).

**Table:** Informed priors used for estimation of the transmission suitability index P for West Nile virus in Israel

Parameter	Distribution means (informed prior)	References
Adult *Culex* mosquito lifespan	10 days (SD = 2)	[41-43]
Extrinsic *Culex–*WNV incubation period	4 days (SD = 1)	[43,44]
Adult *Culex* mosquito biting rate	0.14 per day (SD = 0.02)	[42,45]
Bird lifespan	12 years (SD = 2)	[40]
Intrinsic bird–WNV incubation period	1.5 days (SD = 1)	[40]
Bird–WNV infectious period	6 days (SD = 1)	[40]

SD: standard deviation; WNV: West Nile virus.

Distributions were assumed to be Gaussian.

### Data

Meteorological data were collected from the Israel Meteorological database (https://ims.data.gov.il/), including daily temperature and relative humidity between 1 January 2016 and 30 November 2018, which were available for 97% of the dates.

The human WNV case data were obtained from the Israeli Ministry of Health (https://www.health.gov.il/Subjects/disease/WNF/Pages/default.aspx) and curated to include longitude and latitude coordinates of patient residency. All WNV cases were laboratory-confirmed, either by serum IgM or whole blood RNA tests. Incidence per 100,000 inhabitants was calculated using the population size of each district in 2017 as the denominator (Israel Central Bureau of Statistics; www.cbs.gov.il). Index P estimates, meteorological and case data are available through a (permanent) figshare repository at https://doi.org/10.6084/m9.figshare.c.4584086.v1.

The main results can be reproduced by estimating index P with the freely available Mosquito‐borne Viral Suitability Estimator (MVSE) R package (code as provided in Supplementary Text S2 section 3.13 of [33]), using the Israeli meteorological data as input and the priors listed in the Table. The MVSE is freely available at https://sourceforge.net/projects/mvse/. In the Supplement, we include minimal code that estimates the index P from the Israeli meteorological stations included in this study.

### Analyses and transformations

All case data were transformed using a log_10_(cases + 1) transformation. Area under the curve (AUC) of index P was log_10_-transformed for each district, measured as the average AUC of all stations in the district (December 2016 and 2017 data were excluded for a correct comparison, as December 2018 was missing from the dataset). We note that December 2016 and 2017 had no recorded WNV cases, so the month’s exclusion should not interfere with results.

### Clustering

To measure the similarity between estimated WNV seasonality of different stations, we calculated the Spearman correlation coefficient of index P, humidity and temperature between each pair of stations. We employed the complete-linkage hierarchical clustering algorithm to the created similarity matrices to obtain the division of stations into clusters. In the case of clustering based on both temperature and humidity, we summed the similarity matrices before employing the clustering algorithm.

### Ethical approval

No ethical approval was needed for this study, as all data regarding WNV cases were anonymised (made freely available online through the Israeli Ministry of Health).

## Results

**Figure 1:**
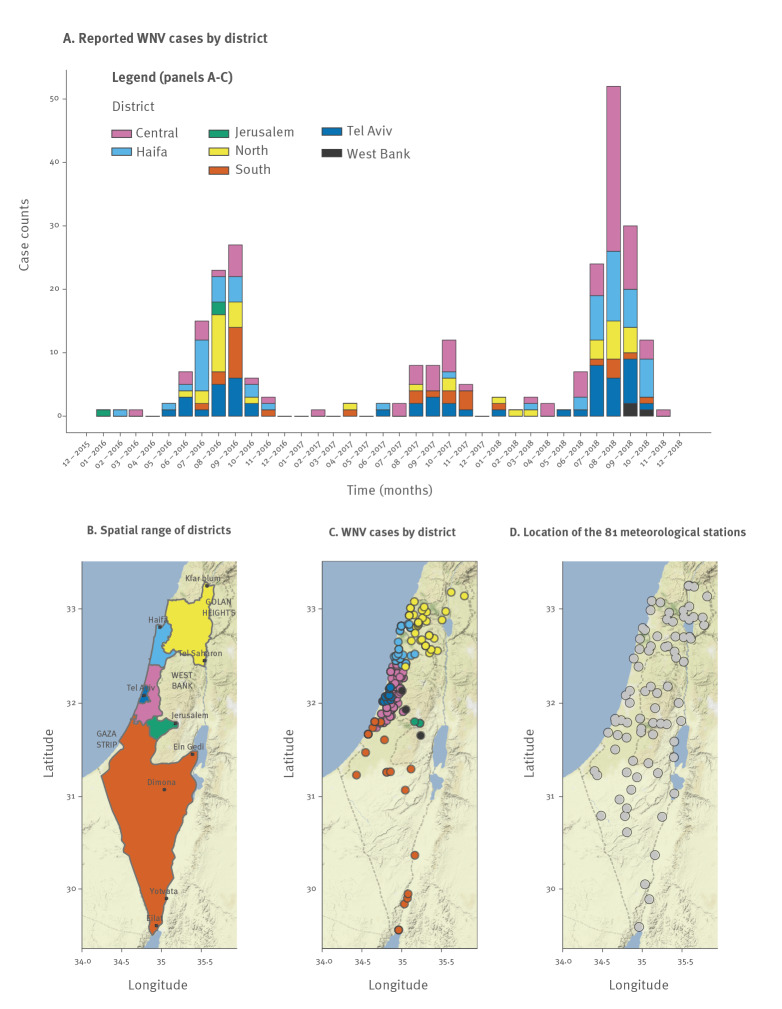
Spatio-temporal description of West Nile virus reports and locations of meteorological stations, Israel, 2016–18 (n =262)

During the period between 1 January 2016 to 1 December 2018, 262 WNV cases were reported, occurring in all the districts of Israel and surrounding regions (Figure 1A-C). The seasonal pattern of cases was similar between the years, with a transmission season roughly between June and November. In these years, incidence peaked between July and September for 2016 and 2018, and a month later in 2017. These patterns were also supported by case data between 2010 and 2015, for which no spatial information was available (Supplementary Figure S1).

WNV: West Nile virus.

Cases in the Golan Heights were considered as part of the Northern district for visualisation.

The highest number of reported cases occurred during 2018, the year in which continental Europe experienced its largest recorded WNV epidemic [19]. That year also saw the second highest number of cases in Israel since 2001, following an outbreak in 2000 comprising more than 400 cases ([38], Supplementary Figure S1). Between 2016 and 2018, the total incidence per 100,000 inhabitants was the highest in the Haifa district (5.72), followed by the Central (3.87), Tel Aviv (3.74), North (3.64), South (2.25) and Jerusalem (0.27) districts. The districts with highest incidence also presented the largest increases between 2016 and 2018 (Haifa, Central and Tel Aviv presented 3.71-fold, 1.5-fold and 1.38-fold more cases in 2018, respectively). The remaining North, South and Jerusalem districts presented a reduced number of cases in the same period (0.94-fold, 0.58-fold and 0 cases, respectively).

### Determinants of West Nile virus transmission potential and seasonality in Israel

**Figure 2:**
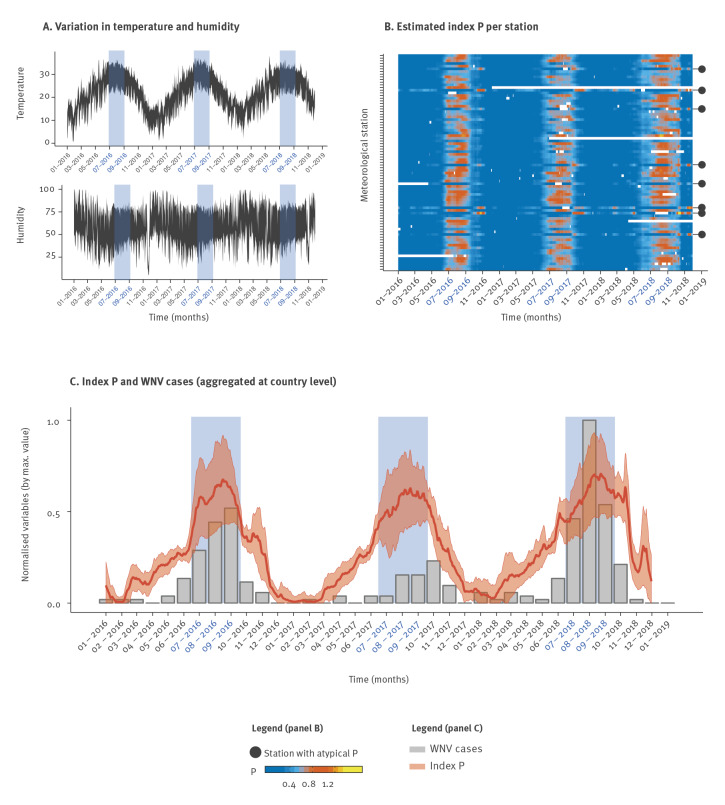
Climatic time series, spatio-temporal description of index P and relationship of index P with case reports of West Nile virus infection, Israel, 2016–18 (n =262)

We obtained daily mean temperature and relative humidity data from 81 geo-located meteorological stations covering six Israeli districts – North, South, Central, Haifa, Jerusalem, Tel Aviv – and surrounding regions (Figure 1D, see Data section). Temperature measurements from all stations presented fluctuations that followed well-defined winter–summer seasonal patterns (Figure 2A). Humidity, on the other hand, presented far more variable fluctuations with no immediately discernible patterns (Figure 2A and Supplementary Figures S2–3).

WNV: West Nile virus.

Panel A presents in black the range between the 2.5 and 97.5 percentiles of the variables as observed across all stations. Blue shaded areas mark the period between July and September.

In Panel B, each row is a time series per day. Stations are ordered alphabetically, from bottom to top. White areas mark days with missing climatic data. Stations with atypical index P are marked with full black circles on the right side of the heatmap.

Panel C shows the total number of WNV reports per month (grey bars), mean and standard deviation of index P per day (orange lines and area). Blue shaded areas mark the period between July and September, during which WNV reports are typically higher. This time window is also marked in blue on the x-axis ticks of all panels.

These daily meteorological series were combined with biological priors for WNV, *Culex* spp. mosquitoes and bird hosts to estimate the index P with a high spatio-temporal resolution between 2016 and 2018. Visualisation of the index P offered a clearer seasonal signal than meteorological variables, with most stations yielding maximal index P between July and September (Figure 2B), the months in which WNV incidence is typically highest (Figure 1A, Supplementary Figure S1). Because the case numbers were small when stratifying by geographical location (or by proximity to meteorological stations), we first addressed the explanatory power of a country-wide aggregated index P (mean across 81 stations) for the sum of notified WNV cases (Figure 2C). We found strong correlations between monthly index P and cases, with Pearson’s correlation coefficients of 0.91 (p = 4 × 10^− 5^), 0.68 (p = 0.016) and 0.9 (p = 2 × 10^− 4^) in 2016, 2017 and 2018, respectively.

Importantly, each year’s correlations between index P averaged over all meteorological stations and the country-wide total cases were consistently higher than the correlations of averaged meteorological variables with cases: temperature yielded correlation coefficients of 0.86, 0.61 and 0.77, whereas humidity exhibited very low correlations coefficients of −0.07, 0.19 and −0.01 (2016, 2017 and 2018, respectively).

### Meteorological heterogeneities and seasonality of West Nile virus transmission potential in Israel

**Figure 3:**
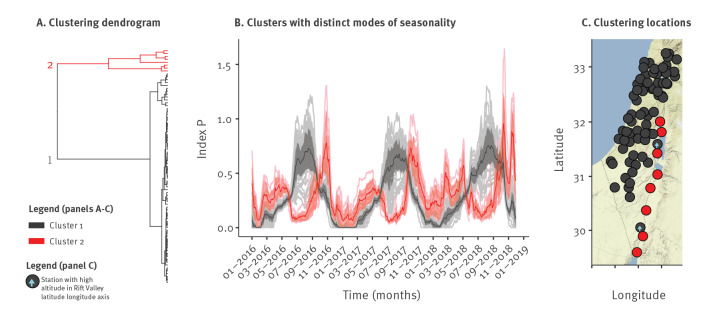
Distinct spatio-temporal modes of estimated West Nile virus seasonality, Israel, 2016–18 (n = 262)

We noted that a minority of stations presented long seasons with high temperature and low humidity (Supplementary Figure S2) while also presenting highly atypical seasonal patterns of index P (Figure 2B, black full circles). To identify and group stations with similar modes of seasonality, we performed a clustering analysis and found two clusters with distinct modes of seasonality (Figure 3A).

Panel A shows the two clusters resulting from the correlation-based clustering analysis of the index P time series (from all 81 meteorological stations).

Panel B shows modes of seasonality according to each cluster, where thinner lines are the index P series of each station coloured according to their cluster, bold lines are the mean and shaded areas the standard deviation.

In panel C, Cluster 2 (red) along the Rift Valley included eight stations: Beit Haarava, Eilat, Ein Gedi, Gilgal, Hazeva, Paran, Sedom and Yotvata, while two stations along the central-southern regions of the Rift Valley (Metzoke Dragot and Neot Smadar) belonged to Cluster 1 and had exceptionally high altitude (marked with blue arrows).

One mode of seasonality had a typical single peak in summer and a trough in winter, while the other mode had two seasonal peaks in spring and autumn and two troughs in summer and winter (Figure 3B). Stations for which the index P estimates followed the second, less common mode, were located at Israel’s centre-southern part of the Great Rift Valley (Figure 3C). Along this region, periods of low humidity and high temperature (hot and dry) coincided with index P’s first trough in summer, whereas extreme low temperature coincided with the second trough in winter (Supplementary Figure S4). The Great Rift Valley is a unique geographical location which includes some of the lowest altitude places on earth. Concordantly, when comparing the altitude of stations producing one or the other mode of seasonality, we found a distinct change in altitude. Whereas the eight stations producing two seasonality peaks are at an average altitude of −168 m (standard error: 74 m), the two closest stations producing one seasonality peak were outside the Rift Valley at altitudes between 300 and 400 m (marked with a blue arrow in Figure 3C).

When applying the same clustering exercise to time series of temperature, humidity and both, we obtained different clustering than we did for index P. Humidity mostly divided the stations along the coastal plain, in western Israel, from those inland (Supplementary Figure S5a). Clustering by temperature, on the other hand, divided the stations in the centre of Israel and surrounding regions from the rest of the stations (Supplementary Figure S5b). Combining both humidity and temperature yielded similar results to the humidity clusters (Supplementary Figure S5c).

### Determinants of the size of West Nile virus epidemics, 2016–2018

**Figure 4:**
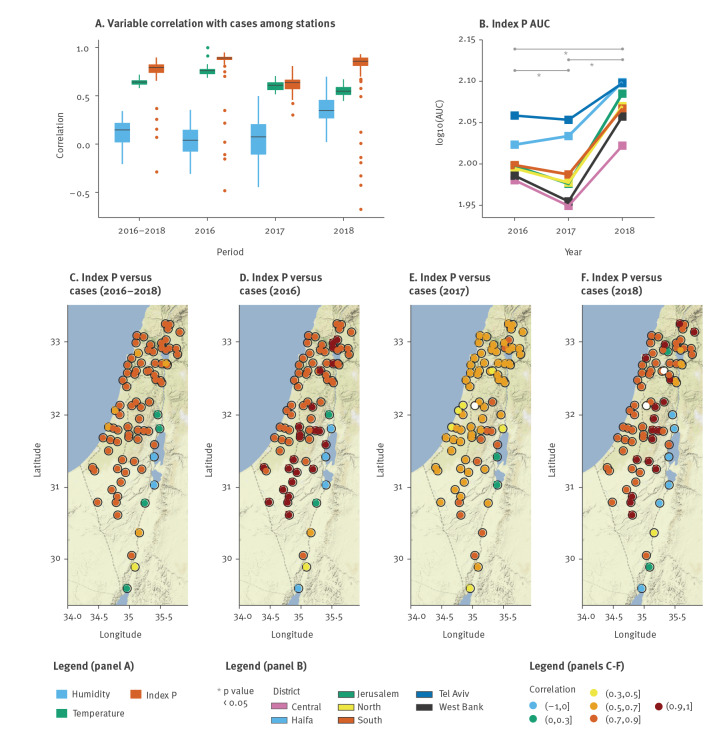
Spatio-temporal determinants of West Nile virus outbreaks, Israel, 2016–18 (n = 262)

AUC: area under the curve; WNV: West Nile virus.

Although temperature and humidity are known to be associated with the incidence of mosquito-borne viral infections [24,35,36], index P yielded consistently higher country-wide correlations to WNV cases in Israel than did either temperature or humidity (Figure 4A). We thus evaluated if yearly differences in index P could also reflect differences in WNV outbreak sizes between the years. This was measured by comparing the AUC of index P for each district per year, resulting in higher AUC values for 2018 than for 2017 and 2016 (paired t-test, p < 8 × 10^− 5^), whereas 2016 had a higher AUC than 2017 (paired t-test, p = 0.034). We also quantified the percentage of time (days) for which the index P was above different thresholds during each of the examined years (Supplementary Figure S6). In accordance with the AUC output, 2018 had consistently longer time windows above any threshold considered (compared with 2016 and 2017). Interestingly, the percentage of time when index P values for 2016 and 2017 were above low thresholds was similar, but when we considered higher thresholds, it changed towards longer time windows in 2016 compared with 2017 (reflecting the AUC difference between the two years). Hence, although yearly aggregated index P may conceal specific differences between the years, such as seasonal shape and duration, it was relevant and reflective of observed differences in WNV outbreak sizes between the years in Israel (cases in 2018 > cases in 2016 > cases in 2017; Figure 4B).

Panel A shows the distributions of Pearson’s correlation coefficients between index P, temperature and humidity of each meteorological station and the country-wide WNV reports, per year and in the whole period 2016–18.

Panel B shows the log_10_ of the area under the curve of the index P per year and district: the mean of P time series is calculated per district, after which the AUC is calculated per year. Statistically significant differences between the years are marked with a grey asterisk.

Panels C to F present Pearson’s correlation coefficients between index P of each station and the country-wide WNV reports (each coloured point), combined through all years and for each year. White points mark stations with missing data.

We also found high correlations (> 0.7) between each station’s index P and case numbers at country-level for most stations located in the coastal plain, the West Bank and the Golan Heights (Figure 4C), concluding that meteorological conditions in stations outside these regions are unlikely to represent the average trends of WNV seasonality in Israel between 2016 and 2018. When breaking down the correlations per year, we found marked differences between the different geographical regions of Israel. High correlations (> 0.7) were found throughout the coastal plain during 2016 and 2018 (Figures 4D and F), while in 2017 these stations presented much lower correlation values (Figure 4E). We note that the patterns of suitability of different stations in 2017 accompanied the overall anomalous behaviour of late onset and low case numbers observed in that year (Figure 1). This yearly mismatch offers a potential explanation for the lower predictive power of index P when averaged across all stations in 2017 (Figure 4A), suggesting that WNV cases in 2017 may have been driven by only a small set of regions, in contrast to other years. Interestingly, this hypothesis would fit with the atypical late onset and small number of cases in 2017. Finally, a pattern of very high correlations (> 0.9) was seen only for the years with largest number of cases (2016 and 2018) in stations in the centre of Israel and aligned in parallel to the Mediterranean Sea (Figures 4D and F), suggesting that these regions may be relevant for the epidemic dynamics of WNV in the country.

## Discussion

In this study we explored the application of a mosquito-borne virus suitability measure, the index P, to characterise the recent spatio-temporal epidemiology of WNV in Israel. Using local meteorological data, we showed that parametrising index P with prior biological knowledge appropriate for *Culex* spp. mosquitoes and a bird reservoir yields an informative predictor of WNV incidence. While temperature and humidity play a major role in the spread of WNV in Israel and may be informative in predicting transmission potential to a certain degree, we have shown that their nonlinear effects on viral and mosquito traits are well captured by the index P. In addition, whereas only one typical mode of WNV seasonality had previously been described in Israel, through our estimations of the suitability measure we have now identified a second, temporally distinct seasonality pattern occurring in the southern parts of the Great Rift Valley in eastern Israel.

There exist two main routes of bird migration through Israel from Asia, Europe and Africa, which are separated by the Negev mountain range [13,32]. Interestingly, one route is along the south-centre-western slopes of the mountain range, matching regions with a WNV mode of seasonality presenting a single yearly peak in our analyses. The other route is to the south-centre-east along the Dead Sea Rift, matching regions where we find the WNV mode of seasonality contains two suitability peaks. Migratory preferences between these routes and susceptibility to WNV vary by bird species [32]. If particular bird species leave or arrive in Israel in periods of high/low transmission potential in either of the regions with different modes of transmission, these findings have significant impact for our understanding of the local epidemiology of WNV, e.g. it may dictate which areas mostly contribute to viral lineage source-sink dynamics in Israel. There are a few ways in which new empirical data and surveillance could be informed by, or even expand on our findings. For example, bird migratory data could be pursued in tandem with estimating WNV transmission potential through the index P, which could lead to identification of key species, timings and regions for local and intercontinental viral dissemination [31,32,38]. Moreover, phylogenetic analysis of WNV isolates can help determine whether the observed case incidence is due to more virulent strains or higher prevalence of the virus driven by higher suitability in certain key time windows and locations identified in our study [31,39]. Interestingly, the 2018 WNV outbreak in Israel was previously investigated phylogenetically to discern whether it resulted from an especially virulent WNV lineage. This hypothesis was ruled out and trends in climatic change were suggested instead but not directly explored [39]. Active surveillance (e.g. mosquito and equine sampling) and control measures (e.g. larviciding) could also be directed to appropriate times and regions with different seasonality modes in order to optimise the cost-effectiveness of interventions [38] and public health preparedness to future outbreaks.

Suitability measures can use meteorological data to estimate the spatio-temporal transmission potential of mosquito-borne viruses and allow exploring local drivers of observed outbreaks. Although the index P was originally developed to suit *Aedes*–human transmission cycles, we here demonstrate that it has high predictive power with regards to reported WNV human cases in Israel. The index can thus be successfully used to characterise the risk of spillover from the WNV zoonotic cycle by changing the biological priors related to the host–pathogen system in question. Although past research has addressed the influence of meteorological variables on some biological processes of *Culex*–WNV, e.g. [27,42], there is currently insufficient characterisation of these relationships to the level that it exists e.g. for *Aedes* mosquitoes. Some of the underlying mathematical formulations defining the relationships between meteorological variables, viral and entomological factors (e.g. mosquito lifespan) have not been adapted in this study to be *Culex*–WNV specific. While this is due to lack of existing empirical data at high resolution that would allow to derive such formulations, it can be recognised as a limitation of the present research. Nonetheless, our results contribute substantially to advances in WNV computational research by demonstrating that WNV transmission potential can be well approximated by relying on empirical evidence from other host–pathogen systems. Hence, our findings provide new ways of contributing to future mechanistic models of WNV transmission. Furthermore, provided that higher resolution empirical knowledge on the direct relationship of meteorological variables and *Culex–*WNV factors is accumulated in the future, incorporation of those into suitability measures such as the index P will be straightforward.

## Conclusion

Our findings in the context of Israel can be seen as a proof of concept that meteorological data can be used to retrospectively reconstruct the local epidemiological history of WNV. Given reliable projections of future trends in meteorological variables, especially in light of climate change, prospective predictions on the likely local, spatio-temporal WNV transmission potential may be possible.

## Supplementary Data

1058000 Supplement
