# Supplementary material for: Characterising West Nile virus epidemiology in Israel using a transmission suitability index
Source: Euro Surveill. 2020 Nov 19;25(46):1900629. doi: 10.2807/1560-7917.ES.2020.25.46.1900629 (PMC7678037; doi:10.2807/1560-7917.ES.2020.25.46.1900629)
Supplement: Supplement [file 1900629_OBOLSKI_Supplement.pdf]

Supplementary Figures and minimal code example

This supplementary material is hosted by Eurosurveillance as supporting information alongside the article "*Characterising West Nile virus epidemiology in Israel using a transmission suitability index*", on behalf of the authors, who remain responsible for the accuracy and appropriateness of the content. The same standards for ethics, copyright, attributions and permissions as for the article apply. Supplements are not edited by Eurosurveillance and the journal is not responsible for the maintenance of any links or email addresses provided therein.

Figures

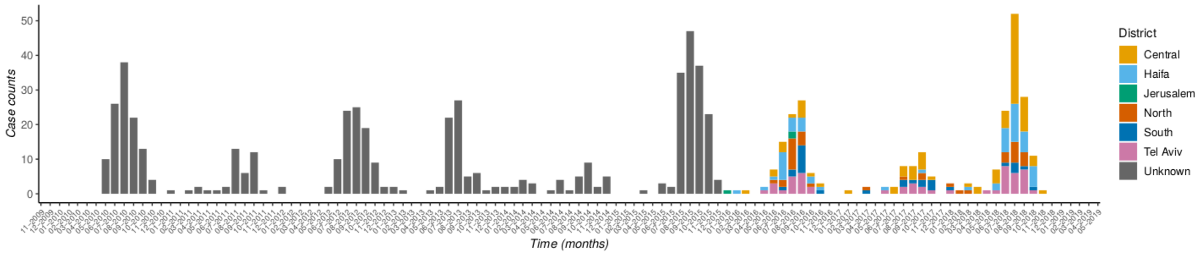

<u>Figure S1:</u> Time series of WNV case reports (2010-2018). Time series of reported WNV case reports by district (in legend). For pre-2016 cases (dark grey), the location of cases was not made available in the surveillance reports. Seasonality in Israel presents a few characteristics that appear stable between the years. For example, the transmission season is roughly between June and November (including a typical incidence peak between July and September), and transmission is low or absent during the winter months.

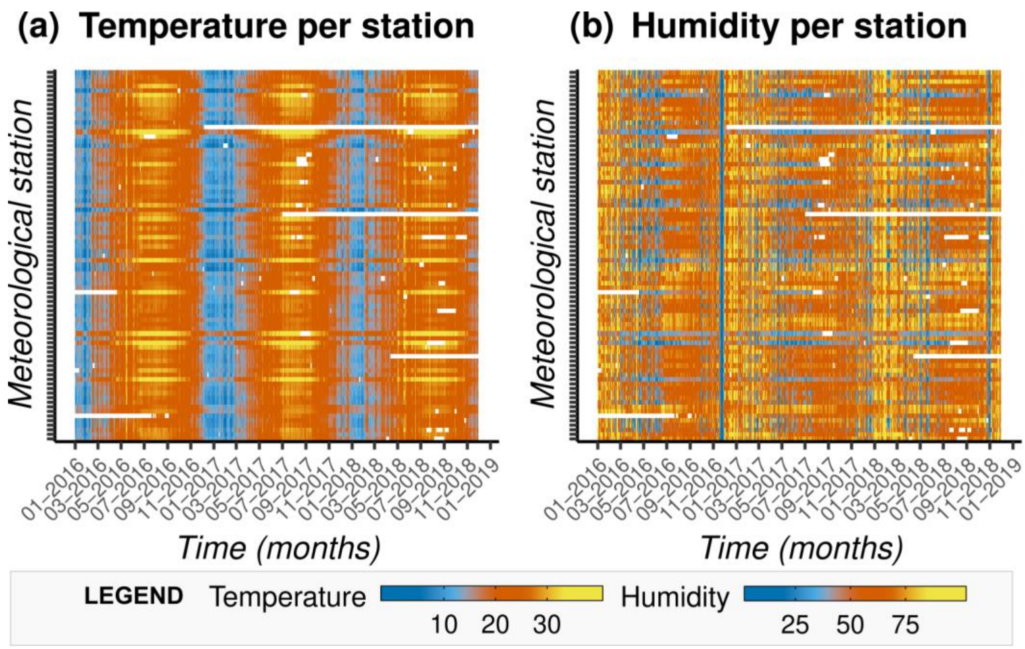

<u>Figure S2:</u> Spatio-temporal description of climatic variables. Heatmaps present (a) temperature (in Celsius) and (b) relative humidity per meteorological station (each row is a time series per day). Stations are ordered alphabetically, from bottom to top. White areas mark days with missing climatic data.

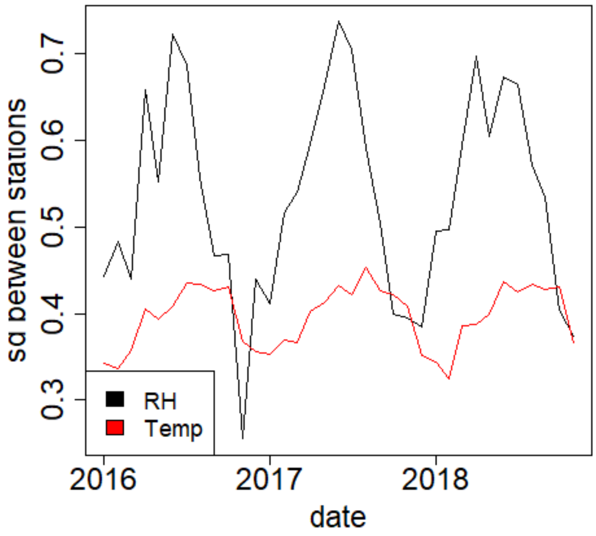

<u>**Figure S3:**</u> **Variation of humidity and temperature among meteorological stations.** After scaling and centering both temperature and humidity time series of each station, we calculate the standard deviations of the average values per month across stations. Temperature is presented in black while humidity in red. The resulting standard deviation between stations per month was higher for humidity then for temperature for approximately 90% of months.

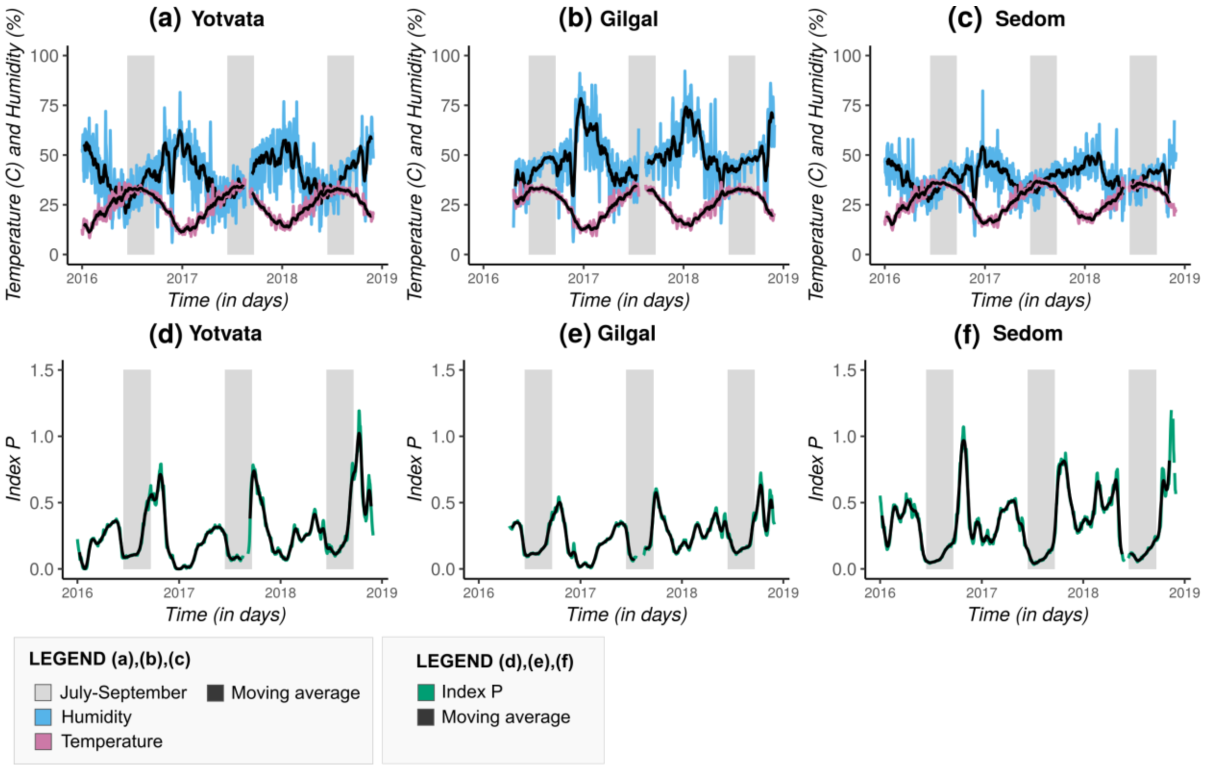

<u>Figure S4:</u> Time series examples of WNV transmission season with two yearly peaks.

(a,d) Time series for humidity and temperature (a) and estimated index P (d) for the meteorological station Yotvata. Grey areas mark the months between July and September, when WNV reports typically peak but the index P is here seen to have a trough due to a temporary mix of very low humidity and maximum temperature. (b,e) The same as (a,d) but for the meteorological station Gilgal and (c,f) for Sedom. These 3 examples are part of cluster 2 in Figure 3 (the cluster presenting two yearly transmission peaks).

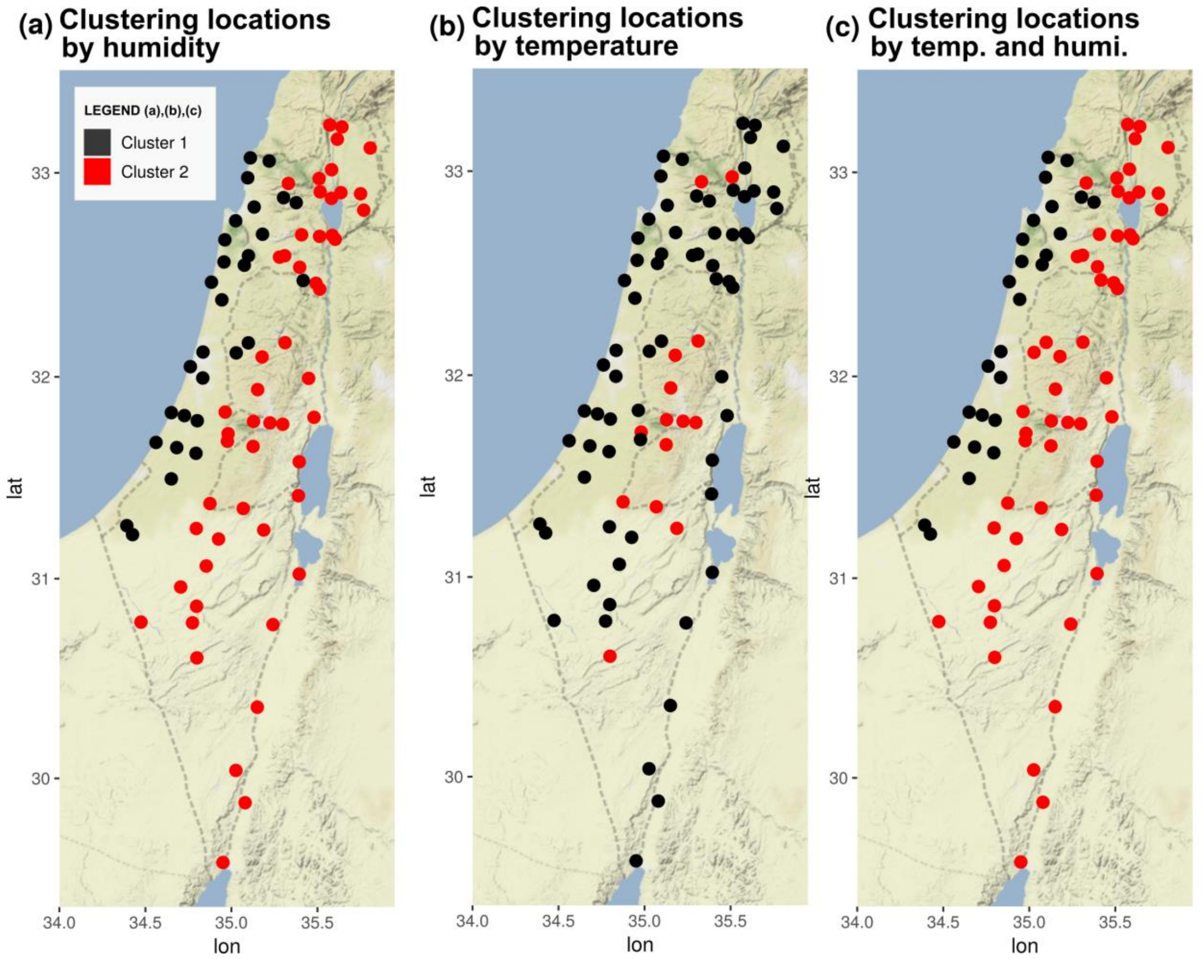

Figure S5: **Correlation-based clustering for humidity and temperature time series.**

Panels present the resulting two clusters from the correlation-based clustering analysis between (a) humidity, (b) temperature and (c) both (from each station. Each cluster groups time-series with similar temporal patterns (see Methods Section). Humidity presents a divide between the regions close to the Mediterranean Sea and the interior of Israel. On the other hand, temperature appears more homogeneous (black cluster found in every district), with the exception of the central and West Bank regions. When using both temperature and humidity for the clustering, stations are grouped similarly to when using humidity; driven by the fact that temperature time series are more homogeneous across Israel (Figure 2a), such that humidity (largely variable, Figure 2b) has a larger effect on the correlation-based clustering.

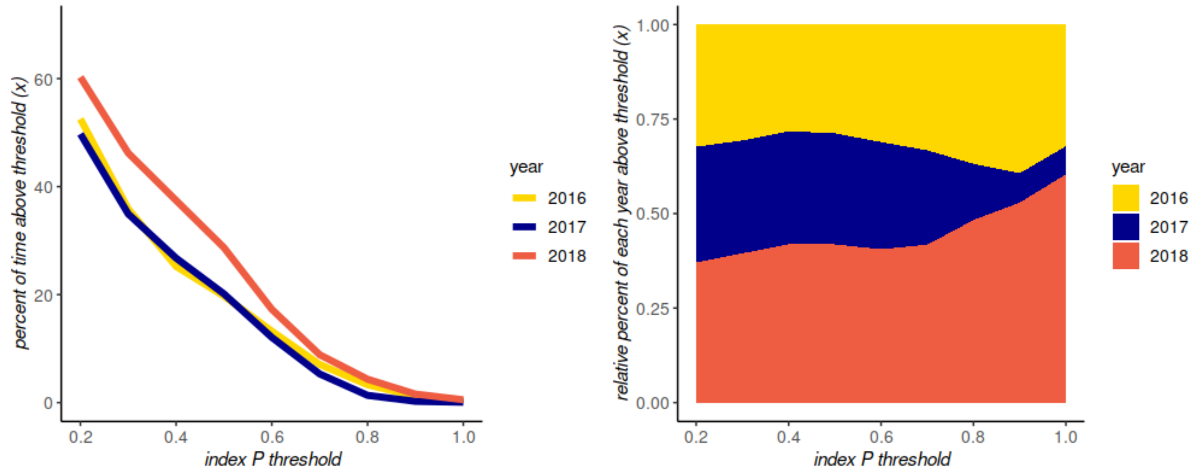

Figure S6: Percent of time of the year the index P remains above particular threshold. (left) For each year, the percent of time that the index P remains above a particular threshold (x-axis) is quantified (y-axis). Percent of time is calculated by counting the days in a year for which the index P was above a particular threshold (x-axis) among all metorological stations. The year 2018 has the highest percent of time for any considered threshold. The years 2016 and 2017 have similar percents of time across the index P thresholds, with 2016 presenting slightly higher percentages. (right) Same output as in the left panel, but presenting the relative percentages between the years. Differences between 2016 and 2017 become evident, in particular as the index P threshold is increased. These results complement the findings in the main text related to Figure 4.

Minimal code example

```
##install MVSE v0.3 :: https://sourceforge.net/projects/mvse/
# install.packages("MVSE_0.3.tar.gz", repo=NULL, type="src")

require(MVSE)

##input file named as downloaded from FigShare repository
load("Climate_Israel20162018_V21.06.2019_TableS2.Rdata") ##loads variable isrdatavg
head(isrdatavg)

##filter data to a single meteo station
station_selected<- "HaifaTechnion"
data<- isrdatavg[isrdatavg$NameFile==station_selected,]

##order data according to date
```

```
data$date<- as.Date(as.character(data$date), format='%d-%m-%Y')
data<- data[order(data$date),]
head(data)

##generally setting priors, bird + culex
MosqLifeExpPrior<- 10
MosqIncPerPrior<- 4
MosqBitRate<- 0.14
HumanLifeExpPrior<- 12
HumanIncPerPrior<- 1.5
HumanInfPerPrior<- 6.0

##note that loading packages such as tidyverse replaces the function 'filter'
##MVSE uses base 'filter' (that is stats::filter) internally
##one can redefine 'filter' to avoid MVSE raising errors
# filter<- stats::filter

##start to use MVSE

setEmpiricalClimateSeries(D=data, NStepSmooth=20)
setOutputFilePathAndTag(station_selected)
dists<- 'gaussian'
setMosqLifeExpPrior(pmean=MosqLifeExpPrior, psd=2, pdist=dists)
setMosqIncPerPrior(pmean=MosqIncPerPrior, psd=2, pdist=dists)
setMosqBitingPrior(pmean=MosqBitRate, psd=0.01, pdist=dists)
setHumanLifeExpPrior(pmean=HumanLifeExpPrior, psd=2, pdist=dists)
setHumanIncPerPrior(pmean=HumanIncPerPrior, psd=1, pdist=dists)
setHumanInfPerPrior(pmean=HumanInfPerPrior, psd=1, pdist=dists)
setHumanMosqTransProbPrior(pmean=0.5, psd=0.01, pdist=dists)

estimateEcoCoefficients(nMCMC=100000, bMCMC=0.5, cRho=1, cEta=1, gauJump=0.75)
simulateEmpiricalIndexP(nSample=1000)
exportEmpiricalIndexP(sep=",") ##creates CSV file with estimated index P time series
plotClimate("climate_output") ## creates PDF with summary of climate time series
```
